# Chronic Nitrogen Deposition Alters Diazotrophic Community Composition, Reduces Biological Nitrogen Fixation, and Restructures Fungal-Diazotroph CO-Occurrence Networks in Deadwood

**DOI:** 10.1007/s00248-026-02849-5

**Published:** 2026-08-01

**Authors:** Shakhawat Hossen, Christina Groß, Friederike Roy, Harald Kellner, Matthias Noll, Werner Borken

**Affiliations:** 1 Institute for Bioanalysis, Coburg University of Applied Sciences and Arts, Friedrich-Streib-Straße 2, Coburg, 96450 Germany; 2 Department of Soil Ecology, University of Bayreuth, Dr.-Hans-Frisch-Straße 1-3, Bayreuth, 95448 Germany; 3 Department of Bio- and Environmental Sciences, International Institute Zittau, TU Dresden, Markt 23, Zittau, D-02763 Germany; 4 Bayreuth Center of Ecology and Environmental Research (BayCEER), University of Bayreuth, Bayreuth, 95448 Germany

**Keywords:** N deposition, *nifH* gene diversity, Biological N_2_ fixation, *nifH* gene abundance, Logs

## Abstract

Biological nitrogen fixation (BNF) by diazotrophs contributes to increasing nitrogen (N) availability in nutrient-poor deadwood during the decomposition process. However, chronically elevated atmospheric N deposition may increase N availability, thereby reshaping diazotrophic community and suppressing BNF. We simulated high N deposition by repeatedly applying ammonium-nitrate solution to deadwood of 13 tree species over 9 years (N addition) and compared diazotrophic community composition and BNF rates with untreated controls. Deadwood N concentrations increased over time in both control and N addition, with N addition resulting in higher N concentrations at the final sampling, although significant treatment effects were detected only in *Tilia* and *Pinus*. Chronic high-N addition was associated with reduced BNF activity, with significant suppression primarily observed in coniferous deadwood, while responses among broadleaved species were weak, variable, or absent. The N addition altered diazotroph richness and community composition by increasing the abundance of *Bradyrhizobium* and by reducing *Methylocapsa* across all tree species. Under N addition, BNF correlated positively with *nifH* gene copy numbers in broadleaved deadwood but negatively in coniferous deadwood. Co-occurrence networks were more interconnected and modular under N addition, with diazotrophs (e.g., *Azospirillum*) central in broadleaved deadwood and fungi (e.g., *Meliniomyces*, *Athelia*) central in coniferous deadwood. Tree clade (coniferous vs. broadleaved) strongly shaped richness and community response, with broadleaved and coniferous species showing distinct patterns.Overall, the largely robust diversity and community composition of diazotrophs and BNF activity under high N addition suggest that moderately increasing N deposition has little influence on fungal deadwood decomposition and the function of deadwood as a carbon pool in forest ecosystems.

**Supplementary Information:**

The online version contains supplementary material available at 10.1007/s00248-026-02849-5.

## Introduction

Deadwood represents a unique biodiversity reservoir in forests and is considered a nitrogen (N)-limited substrate for bacteria and fungi involved in its decomposition [1, 2]. Recent studies have highlighted that diazotroph bacteria within decomposing deadwood actively fix atmospheric N_2_, compensating for N limitation and contributing to N enrichment in deadwood as the decay stage progresses [2, 3]. Anthropogenic N emissions have markedly increased global N deposition through rapid expansion of agricultural, industrial, and urban activities, with deposition rates rising 3-fold over land since 1850 and continuing to accelerate [4]. Chronic N deposition affects forest ecosystems by altering soil carbon (C) cycling and wood decomposition, leading to high N retention in the forest floor, which can enhance C accumulation in tree biomass but may also result in C loss in N-saturated forests, impacting overall C sequestration [5, 6]. Understanding diazotroph responses to N enrichment is critical because these microorganisms sustain BNF - a key atmospheric N input pathway yet their response to elevated N deposition remains paradoxical and poorly integrated into biogeochemical or C models for forest ecosystems [7, 8].

Diazotrophs can fix atmospheric N_2_ into ammonia via the dinitrogenase complex, which is encoded by the *nif* gene family [9]. The *nifH* gene is crucial for the dinitrogenase complex and has been found to be a reliable biomarker for studying diazotrophic diversity [10, 11]. In the decaying deadwood, diazotroph bacteria form mutualistic associations with wood-inhabiting fungi, driven by severe N limitation in this substrate [12]. Wood-inhabiting fungi degrade lignocellulose and supply diazotrophs with readily available carbohydrates and micronutrients such as molybdenum, an essential nitrogenase cofactor, while BNF by diazotrophs in turn alleviates the severe N limitation that constrains fungal growth [12–14]. The strength of this C for N exchange can vary with decay stage, type of wood rot, and host tree species [12, 15, 16]. BNF activity is generally higher in advanced stages of brown-rotted wood, while the composition of *nifH* communities is shaped by both tree species and decay class [13, 17]. Recent metagenomic and metatranscriptomic studies have revealed a diverse range of diazotrophic taxa inhabiting deadwood, predominantly within the Proteobacteria (e.g., *Rhizobium*, *Bradyrhizobium*, *Sphingomonas*) and Actinobacteria [18, 19]. These organisms are particularly active when diverse labile C sources are abundant, but N remains limiting. Their metabolic capabilities not only support their own growth but also facilitate the colonization and activity of other microbial decomposers by gradually enriching the substrate with N relative to C. The composition of diazotrophic communities is influenced by wood traits such as phosphorus, molybdenum, magnesium, glucose concentration, and the C: N ratio of the respective tree species [2, 20]. How diazotroph communities in deadwood of various tree species respond in the long term to chronic atmospheric N deposition remains largely unknown.

Deadwood acts as a N sink by absorbing and retaining N from the atmosphere during decomposition [21, 22]. Additionally, deadwood can serve as an N source through the leaching of dissolved organic N [23], thereby increasing the N content in the soil beneath deadwood [24]. The amount of C and N in deadwood can vary greatly depending on several factors, including tree species, decay stage and the surrounding environmental conditions [25–27]. During the progression of deadwood decomposition, C concentration decreases and N concentration increases, as microbes utilize the C-rich woody compounds, release CO_2_, and enrich N by their microbial biomass and accumulation of microbial necromass [28, 29]. Furthermore, the decomposition process and N turnover in deadwood can also be influenced by tree species-specific characteristics or the tree clade, such as macro- and micronutrient contents and the physical structure of the wood [30].High atmospheric N deposition increases the bioavailability of N, altering the overall structure and function of ecosystems [31]. N deposition is considered to be one of the most important drivers of shifts in the biodiversity in terrestrial ecosystems [32]. Since the 1990s, levels of anthropogenic N deposition have been steadily declining in Europe and North America. However, current deposition rates remain high compared to pre-industrial levels, with values ranging between 5 and 20 kg N ha⁻¹ year⁻¹, depending on region and land-use intensity [33, 34]. In areas with high agricultural activity and industrial emissions, N deposition can still reach levels > 15 kg N ha⁻¹ year⁻¹ in forest ecosystems, continuing to influence nutrient dynamics and growth of forests [35]. Increased N deposition leads to shifts in the abundance of deadwood-dwelling and decaying species across trophic levels, including fungi, bacteria, insects, and mammals, amplifying the decomposition of deadwood [36, 37].

Similar to changes in species abundance, adaptation of the microbial community composition to increased N deposition could also affect decomposition processes of deadwood. It has been shown that N deposition selectively affects N-limited gymnosperm deadwood by enhancing fungal metabolic activity rather than by fundamentally restructuring decomposer communities [16]. In highly N-limited habitats, fungal communities showed resistance to N addition, maintaining ecosystem functions through adaptation and functional redundancy [38]. Beyond deadwood, N deposition has also altered the composition of active fungal communities in forest floors [39] and has been shown to suppress N fixation rates and reduce diazotrophic community abundance in soils [40]. Additionally, previous studies have reported that N deposition affects fungal and bacterial diversity and alters decomposition dynamics in leaf litter within temperate and boreal forests [41–44].

N-fixing bacteria play a complementary role in deadwood decomposition by making N bioavailable, while being dependent on fungal activity for deadwood colonization and C supply [46]. This fungal-bacterial interaction is evident in network analyses, revealing ecological links between these taxa [13, 19]. N-fixing bacteria, particularly from the orders *Burkholderiales* and *Rhizobiales*, form networks with wood-inhabiting fungi to support decomposition processes by exchanging N for C resources [13, 20]. These microbial interactions in deadwood range from mutualistic to antagonistic and are influenced by decay stage, wood chemistry, and environmental conditions [19, 45, 46]. The impact of N addition on fungal community composition was shown to be minor in both the early [38] and advanced stages of deadwood decay [16]. Mutualistic interactions between defined fungal and diazotrophic taxa have been documented in deadwood ecosystems [13, 18]. However, it remains unclear how chronic N enrichment affects the stability of these fungal–diazotroph associations that govern BNF in deadwood.

In this study, we investigated the long-term effects of N addition on diazotrophic communities during deadwood decomposition across 13 temperate tree species exposed to nine years of elevated N input. The 13 species were selected to represent common temperate European forest trees. This composition mirrors other large-scale deadwood studies: the BELongDead experiment used the same 13 species across ~ 10 years [47]. We hypothesize, first, that chronic N addition alters diazotrophic diversity, reduces *nifH* gene abundance and BNF rates, and disrupts fungal-diazotroph associations by reducing co-occurrence and connectivity in microbial networks. Second, we hypothesize that these effects are not uniform across the 13 tree species but instead vary with tree species and between coniferous and broadleaved clades.

## Experimental Procedures

### Study Site and Design

The N experiment was carried out within the BELongDead experiment [30, 47], which is part of the Biodiversity-Exploratories research platform described in [48]. It was conducted across five forest plots, composed of *Fagus sylvatica* (> 80%), *Acer* spp., *Carpinus betulus*,* Fraxinus excelsior*, and *Tilia* spp. (together < 20%), in the Hainich National Park (51.097324 N 10.458212E), in Thuringia, Central Germany. In the five forest plots, mean annual temperature was 8.0–9.0 °C, and annual precipitation was 570 to 620 mm during the experimental period. The distances between plots ranged from 0.4 to 28.7 km as described in a previous study [30].

The experiment consisted of 13 logs per plot, belonging to 13 different temperate tree species (nine broadleaved: *Acer* spp, *Betula pendula*, *Carpinus betulus*, *Fagus sylvatica*, *Fraxinus excelsior*, *Populus* spp., *Prunus avium*, *Quercus* spp., *Tilia* spp.; four coniferous: *Larix decidua*, *Picea abies*, *Pinus sylvestris*, and *Pseudotsuga menziesii*), which were harvested from the same region in Thuringia. Further details on the logs are provided in [16]. Two sections, each approx. 30 cm in diameter and 40 cm in length, were cut from each log per plot (in total 130 log sections), and then placed individually in plastic boxes (Euro packaging box 60 × 40 × 32 cm, Auer GmbH Amerang, Germany) to prevent contact with soil and input of soilborne N. In addition, tile pieces were placed underneath the log sections to prevent fungal growth from the soil, improve aeration, and ensure that the log sections did not remain in standing rainwater. To minimize the accumulation of precipitation inside the plastic boxes, drainage was facilitated by small holes in the bottom of the boxes. For each log section pair per tree species and plot, one half (*n* = 65) was treated with NH_4_NO_3_ solution while the other half (*n* = 65) served as a control without N addition. To simulate N deposition, 5 mL of 40 g L^− 1^ NH_4_NO_3_ was uniformly hand-sprayed onto each log each month, which simulates an additional annual N deposition rate of about 40 kg N ha^− 1^ year^− 1^ until 2020 (9 years of continuous N addition), while the control was sprayed accordingly with 5 mL of deionized water. Regional background deposition in the study area was ~ 15 kg N ha⁻¹ yr⁻¹ [49]. A detailed description of plot layout, dosing schedule, and sampling is provided in [38].

### Sample Collection

The experiment started in September 2011, and wood samples were first collected at the onset of the experiment (September 2011) and subsequently after two years (October 2013) to monitor early decomposition dynamics. Sampling was conducted using a cordless drill (Makita BDF 451) fitted with a 10 mm diameter, 450 mm long wood auger. Wood shavings were obtained by drilling radially through the entire log (~ 30 cm) with the wood auger and collected in sterile sample bags. Thus, the samples represent a mixture of sapwood and heartwood, while bark was removed before drilling. To prevent cross-contamination between samples, the auger was sterilized after each drilling by immersion in alcohol, brief flaming, and wiping with ethanol [38]. The last sampling campaign was conducted in September 2020. As most of the logs had reached late stages of decomposition by this time, deadwood mass was determined to assess mass loss [16]. Representative subsamples were taken for various analyses, including water content, chemical composition, respiration, BNF, and microbial communities. Samples were transported to the laboratory at cooled conditions and were stored either at -80 °C for molecular biological analysis or at 2 °C for all other analyses. The samples stored at 2 °C were processed within one week.

### Biological Nitrogen Fixation

Deadwood samples were gently cleaned by removing litter and moss tissues and then chopped into small pieces < 1 cm in diameter. To determine the BNF rate, deadwood samples were divided into two subsamples. One subsample was dried at 60 °C for 48 h and used to determine the natural ^15^N abundance in deadwood (see below). The second subsample was used for ^15^N_2_ incubation, with 1–5 g of fresh weight placed in a 25.6 mL crimp vial. The fresh weight of deadwood differed among tree species because of distinct wood densities and decay stages. To prevent microbial activity from being inhibited by insufficient water content, a minimum water holding capacity of 60% was achieved by adding 0.5 mL of deionized water to each vial. The samples were pre-incubated at 20 °C in the dark for four days. For ^15^N_2_ incubation, the vials were sealed with butyl stoppers, crimped with aluminum caps, and then flushed for 15 min with a gas mixture, consisting of 8% oxygen (O_2_), 30% N_2_, and 62% helium (Riessner-Gase GmbH, Lichtenfels, Germany). Thereafter, 4 mL of purified (see below) ^15^N_2_ (99.1%, Sigma-Aldrich Chemie GmbH, Taufkirchen, Germany) was injected into each glass vial using a gas-tight syringe. The samples were incubated for 72 h at 20 °C in the dark. To stop BNF, the vials were opened and the samples were immediately dried at 60 °C for 48 h until mass constancy. Dried deadwood samples were ground using a ball mill (MM400, Retsch GmbH, Haan, Germany) as described in [50].

Incubated and respective non-incubated samples were analyzed for δ^15^N signatures and N concentrations at the Centre for Stable Isotope Research and Analysis, Göttingen, using an element analyzer (Euro EA 3000, EuroVector S.p.A., Milano, Italy), coupled to a Delta C isotope mass spectrometer with a ConFlo III interface (Thermo Electron, Bremen, Germany) or an element analyzer (Euro MA 3000, EuroVector S.p.A., Milano, Italy), coupled to a Delta C isotope mass spectrometer with a Con-FloII interface (Thermo Electron, Bremen, Germany) for natural ^15^N abundance and ^15^N enriched samples, respectively. Values of δ^15^N are reported vs. the natural isotopic abundance (^14^N/^15^N) of N_2_ in the atmosphere (0‰, 0.3663 atom%), using certified reference materials and working standards of known isotopic composition. The δ^15^N signatures of deadwood samples were converted into atom% ^15^N as follows:

1 $$\:{R}_{S}=\:\:{R}_{air}\:\times\:(\delta\:{}^{15}N/1000+1)\:\:\:$$

2 $$\:\mathrm{a}\mathrm{t}\mathrm{o}\mathrm{m}\mathrm{\%}\:{}^{15}N=\left(\:\frac{{R}_{s}}{{R}_{s}+1}\right)\times\:100$$

where *R*_*s*_ is the ratio of ^15^N/^14^N in a deadwood sample, *R*_*a.i.*r_ is the standard ratio for atmospheric N_2_, which is 0.0036765.

To calculate the BNF rate, it was necessary to determine the ratio of ^15^N_2_ to total N_2_ in the headspace (HS) for each vial. This was determined based on the amount of ^14^N_2_, ^15^N_2_, and gas pressures before and after addition of ^15^N_2_. The calculations revealed ^15^N signatures ranging from 30 to 40 atom% for individual vials. BNF rates in deadwood (µg N g^− 1^ d^− 1^) were calculated using the ^15^N signature (atom%) of incubated and non-incubated (natural abundance, NA) samples as follows:

3 $$\:BNF=\frac{N\:\times\:\:\left(atom\%{}^{15}{N}_{enriched}\:-\:atom\%\:{}^{15}{N}_{NA}\right)}{DW\times\:t\times\:HS\times\:100}$$

where N is the amount of N in deadwood (µg), ^15^N_enriched_ is atom% ^15^N in the incubated deadwood sample, ^15^N_NA_ is the atom% ^15^N in the non-incubated deadwood sample (natural ^15^N abundance), DW is the dry weight of deadwood (g), t is the incubation time (d), and HS is the ratio of ^15^N_2_ to total N_2_ in the headspace of the vial. After subtracting the blank (autoclaved samples, see below), a few negative BNF values (< 0.02 µg N g⁻¹ d⁻¹) were calculated, which were set to zero.

The purification of the ^15^N_2_ gas was necessary to remove potential contaminations by ^15^NH_3_ and ^15^NO_x_ (see [50] for further details). To determine residual contamination of deadwood by the purified ^15^N_2_ gas, four deadwood samples were autoclaved at 121 °C and 1.2 bar for 20 min. The autoclaved samples were incubated with purified ^15^N_2_ gas under the same conditions as the non-autoclaved deadwood samples. The enrichment in the δ^15^N signature in the autoclaved deadwood samples was negligible (0.001 µg ^15^N per ml ^15^N_2_).

### DNA Extraction and Quantitative PCR (qPCR)

The DNA from deadwood samples was isolated using the Quick-DNA Fecal/Soil Microbe Miniprep Kit (Zymo, California, USA) according to the manufacturer’s instructions. To quantify the number of diazotroph gene copies, the *PolF* and *PolR* primer set [51] was used for qPCR as explained in [2]. Briefly, the reactions were performed in 96-well plates using the CFX96™ Real-Time System (Bio-Rad, Feldkirchen, Germany), with nuclease-free master mix blanks serving as the negative control. The number of *nifH* gene copies was calculated by comparing the PCR-cycle threshold values to dilutions of genomic DNA extracted from *Azotobacter vinelandii* (DSM 2289). Cell counts of *A. vinelandii* were carried out by using a Petroff counting chamber (Paul Marienfeld GmbH, Germany) as the basis for *nifH* gene copy numbers after subsequent nucleic acid extraction. For each set of reactions, a five-point tenfold serial dilution of *A. vinelandii* genomic DNA (10-100000 fg, ranging from 5 × 10^2^ to 5 × 10^7^ *nifH* gene copy numbers) was run in triplicate to establish the standard curve.

The qPCR reactions were carried out in 10-µL assays comprising 5 µL SYBR^®^ Green Supermix (Bio-Rad, Feldkirchen, Germany), 0.5 µL of each primer (*PolF* and *PolR*) at a concentration of 2.5 µM, 3 µL of sterile and nuclease-free water (Carl Roth GmbH), and 1 µL of either 1:10 diluted DNA-extract, ten-fold diluted standard DNA or 1 µL water for no template control. The cycling conditions were as follows: initial denaturation at 94 °C for 5 min followed by 40 amplification cycles of 95 °C for 1 min (denaturation), 55 °C for 1 min (annealing), 72 °C for 1 min 30 s (extension), and a final elongation step at 72 °C for 5 min. To check the specificity of the product and the possibility of primer dimer formation, the experiment was completed with a melting analysis starting from 65 °C to 95 °C with temperature increments of 0.5 °C and a transition rate of 5 s. The amplicon sizes were checked by electrophoresis on a 1.5% agarose gel. To ensure that there were no inhibitors in the qPCR assays, multiple dilutions (non-diluted, 1:10, and 1:100 dilution) were run simultaneously, and no PCR inhibition was observed. As *A. vinelandii* has a single *nifH* gene copy in its genome as listed in the NCBI nucleotide sequence database, the *nifH* gene copy numbers were calculated and set in relation to the dry mass of deadwood.

### The nifH Gene-Based Amplicon Sequencing and Bioinformatics Analysis Pipeline

The *nifH* gene of each sample was independently amplified three times using the PCR reaction as described above, and the resulting amplicons of each of the three replicates were pooled. Pooled *nifH* gene amplicons were sent to an external sequencing provider (LGC Genomics, Berlin, Germany). Illumima-based sequencing adapters were ligated to both ends of the amplicons to create libraries and only qualified libraries were selected for high-throughput sequencing using the Illumina Miseq sequencer platform. The samples were then multiplexed and run on a paired-end (2 × 300 bp) sequencing run. In this study, Illumina-generated paired-end sequences were merged using BBMerge v34.48 [52], and the resulting combined sequences were subjected to quality filtering using the DADA2 algorithm [53]. This algorithm was used to generate amplicon sequence variants (ASVs) by resolving amplicon-sequencing errors. Non-target-length sequences were removed from the sequence table, and only sequences with a length between 250 and 400 bp were retained. To identify and classify organisms, a customized *nifH* gene reference database compiled by TaxADivA [11] was used. A total of 4,470,563 quality-checked *nifH* gene sequences were obtained from 130 deadwood samples, and 6,043 rarefied ASVs were obtained. The analysis revealed 82 diazotrophic genera across all samples. Fungal sequencing dataset was obtained from the same nucleic acid extracts as explained in [16].

### Construction of Co-Occurrence Networks of Fungal and Diazotroph Communities

The absolute abundance matrices for each dataset were imported into the CoNet ensemble app, which is available in Cytoscape version 3.9.1 [54]. In this app, the rows represent ASVs, and the columns represent logs. The same settings were applied to both the control and N addition datasets. To filter the data and address sparsity issues and false correlations, the ASVs were filtered based on their occurrence across samples using the minimum value, keeping the sum of filtered rows (row minimum occurrence = 12 for broadleaved and 6 for coniferous). This filtering step helped to minimize issues caused by the presence of double zeros when computing similarity or distance coefficients using species presence-absence or abundance data [54, 55]. To reduce compositionality issues arising from different sampling efforts, the data were normalized by column. Four correlation and dissimilarity metrics were selected for analysis: Spearman, Pearson, Bray-Curtis dissimilarity, and Kullback-Leibler dissimilarity, as described in [56]. The correlation thresholds were set to retain 1000 edges, which consisted of the top positive and negative correlations, supported by all four metrics (score = mean). The edge significance was tested through 1000 permutations and bootstraps using the “brown” method. Only the edges with merged p-values < 0.05 after Benjamini- Hochberg correction (q-value) were retained.

### Statistical Analyses

The statistical analyses, including both comparisons between treatments (C = control and N = N addition), tree species, and tree clade (broadleaved vs. coniferous) were conducted using two software programs: R (v4.2.1) [57] and PAST version 2.17 [58]. The datasets were evaluated for normality using the Jarque-Bera test, and for equality of group variances using the F-test (for two datasets) and Levene’s test (for more than two datasets). Mass loss of deadwood was used from our previous study [16]. Overall effects of N addition and either tree species or tree clade (broadleaved vs. coniferous), together with their interactions on diazotroph richness, N concentration, BNF, and *nifH* gene copy numbers were evaluated using separate two-way ANOVA implemented in PAST. Following these analyses, the same four variables were compared between the control and N addition treatments within each tree species using paired sample t-tests or, Wilcoxon signed-rank tests when paired differences did not meet the normality. Diazotrophic community composition was analyzed using separate two way permutational multivariate analyses of variance (PERMANOVA) for N addition × tree species and N addition × tree clade (broadleaved vs. coniferous). Both analyses were based on Bray–Curtis dissimilarities calculated from relative-abundance data with 999 permutations. Non-metric multidimensional scaling (NMDS) was used for visualization, and all analyses were performed using the “Vegan” package in R [59]. Spearman’s rank correlations were used to evaluate relationships among BNF, *nifH* gene copy numbers, fungal richness, diazotroph richness, and deadwood mass loss. Correlations were calculated separately for control and N-addition treatments across all samples and, within each treatment, for broadleaved and coniferous deadwood. Data from the final sampling year (2020) were used for all analyses, figures, and tables, except for N concentration data presented in Table 2, which included measurements from three time points (2011, 2013, and 2020). A two-way ANOVA was used to evaluate the effects of year (2011, 2013, 2020), treatment (control vs. N addition), and their interactions on N concentration.

## Results

### The Diazotrophic Diversity and Community Composition

**Fig. 1:**
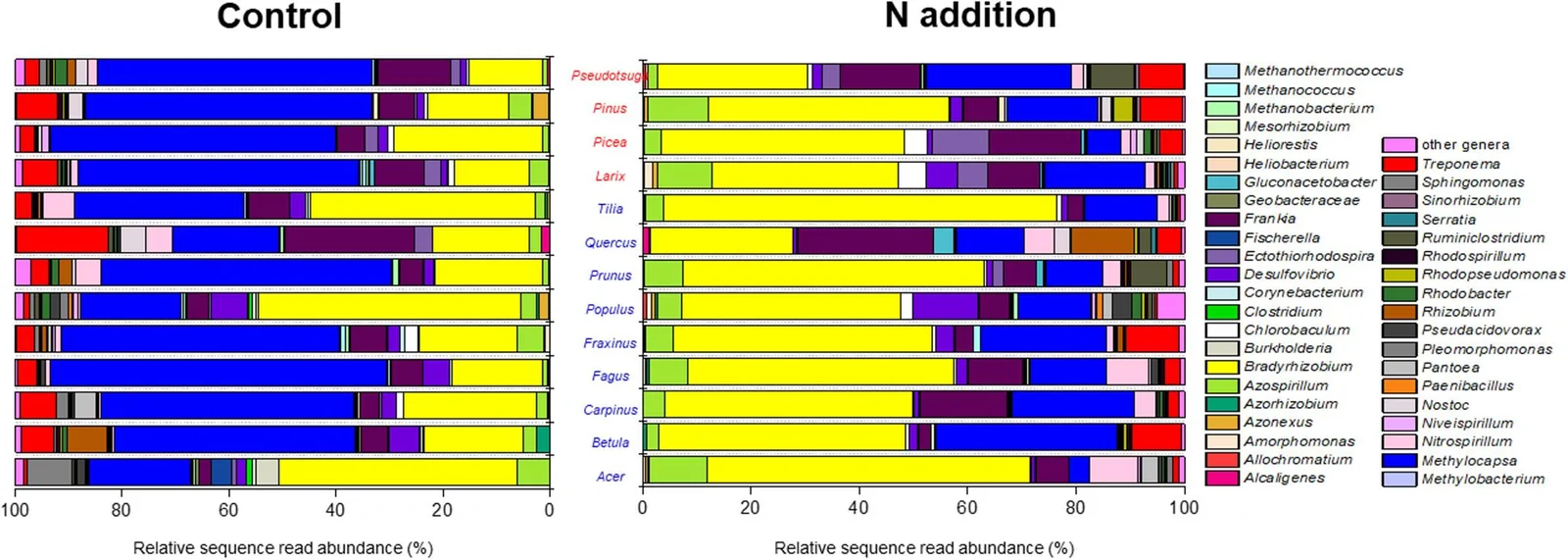
Mean relative sequence read abundances of diazotrophic genera from two deadwood treatments (control, N addition) of 13 tree species after nine years of decomposition. Genera with a proportion < 0.1% in all samples are summarized as “other genera”

**Fig. 2:**
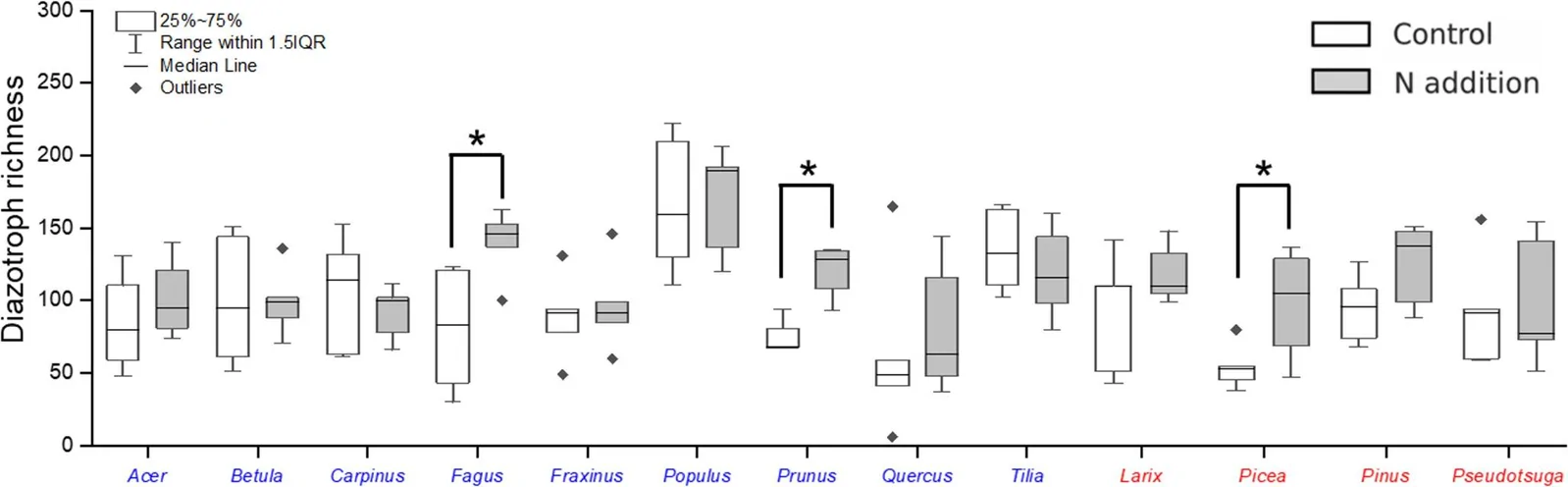
Diazotrophic richness in two deadwood treatments (control, white; N addition, grey) of thirteen tree species after nine years of decomposition. Deadwood of broadleaved and coniferous tree species is labeled in blue and red, respectively. Significant effects of N addition within tree species are indicated by asterisks (*P* < 0.05)

The distribution of diazotrophic taxa was dominated by members of the genera *Methylocapsa* (43% relative sequence read abundances), *Bradyrhizobium* (25%), and *Frankia* (8%) in the control treatment (Fig. 1). The N addition significantly (*P* < 0.05) altered the relative abundance of the most prominent diazotrophic taxa across all tree species (Figure S1). In particular, N addition increased the relative abundance of the genus *Bradyrhizobium* from 25 to 46% and decreased the relative abundance of *Methylocapsa* from 43 to 17%. Moreover, *Burkholderia*, *Corynebacterium*,* Serratia*, and *Sinorhizobium* were not detectable across all tree species in the N addition (Fig. 1). In the tree species model, diazotroph richness was significantly influenced by both N addition (*F*_N addition_ = 7.40, *P* = 0.007) and tree species (*F*_tree species_ = 4.50, *P* < 0.001), whereas the interaction between N addition and tree species was not significant (*F*_N addition x tree species_ = 1.02, *P* = 0.431) (Table S1). Specifically, diazotroph richness increased significantly under N addition in *Fagus*, *Prunus*, and *Picea* logs *(P* < 0.05), whereas the remaining ten species (*Acer*, *Betula*, *Carpinus*, *Fraxinus*, *Populus*, *Quercus*, *Tilia*, *Larix*, *Pinus*, *Pseudotsuga*) showed no significant differences (Fig. 2). Similarly, in the tree clade model, N addition had a significant effect on diazotroph richness (*F*_N addition_ = 5.54, *P* = 0.020), whereas tree clade had no significant effect (*F*_tree clade_ = 1.78, *P* = 0.184). The interaction between N addition and tree clade was not significant (*F*_N addition x tree clade_ = 0.84, *P* = 0.360) (Table S1). 

**Fig. 3:**
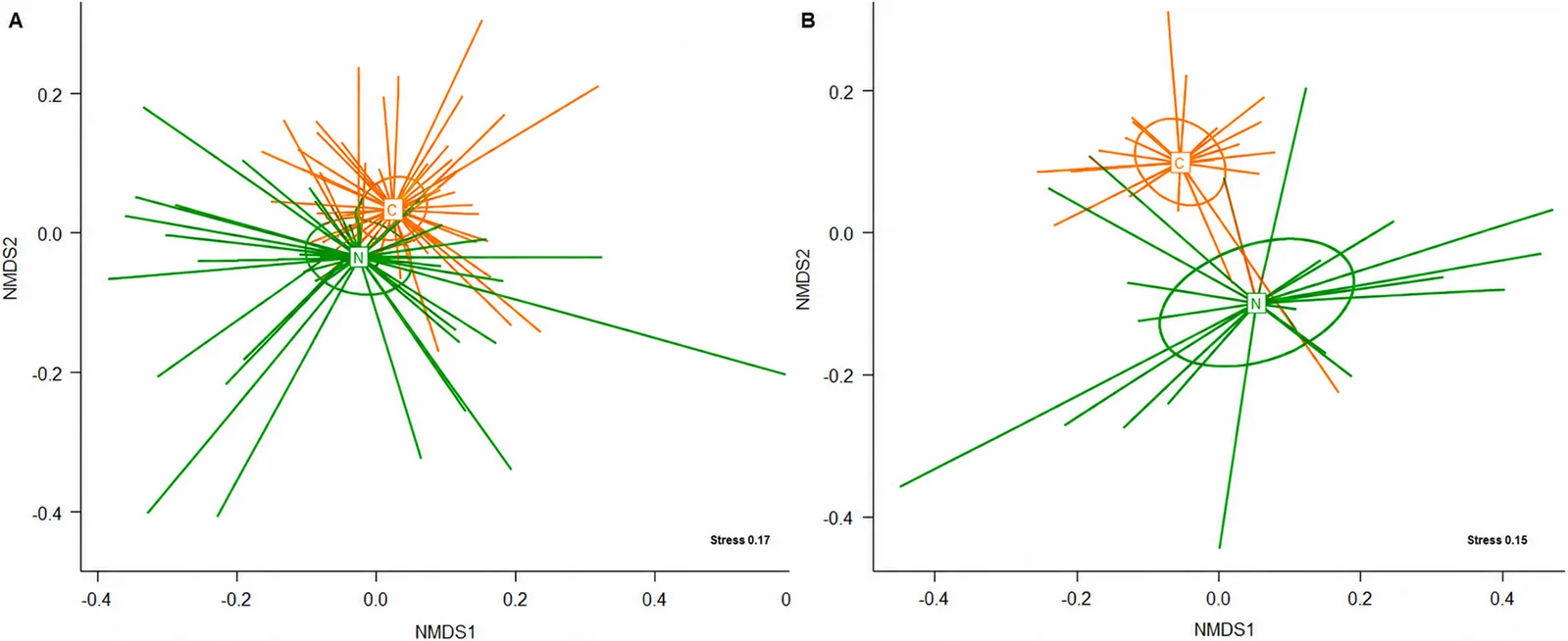
Non-metric multidimensional scaling (NMDS) ordination displaying diazotrophic community structure for (A) broadleaved and (B) coniferous in relation to treatment (orange = control, green = N addition,). Each emanating line connects an individual sample to the centroid of its corresponding treatment group, while the ellipses summarize the dispersion of samples around each group centroid

Both N addition (*F*_N addition_ = 3.40, *P* < 0.001) and tree species (*F*_tree species_ = 1.93, *P* < 0.001) significantly affected diazotroph community composition, but their interaction was not significant (*F*_N addition x tree species_ = 1.01, *P* = 0.426) (Table 1). Similarly, in the tree clade model, diazotroph community composition was significantly influenced by both N addition (*F*_N addition_ = 3.19, *P* < 0.001) and tree clade (*F*_tree clade_ = 3.34, *P* < 0.001). In this case, however, the interaction between N addition and tree clade was significant (*F*_N addition x tree clade_ = 1.40, *P* = 0.041) (Table 1), indicating that the N addition effect was tree clade (broadleaved vs. coniferous) dependent. This effect was more pronounced in coniferous than in broadleaved deadwood (Fig. 3). 

**Table 1:** Effects of N addition, tree species, and tree clade (broadleaved vs. coniferous) on diazotrophic community composition based on separate two way permutational multivariate analyses of variance (PERMANOVA). Significant variables *P* < 0.05 are shown in bold

	Effect	df	Sum of squares	*F*	*R*²	***P***
*N* addition × tree species	*N* addition	1	1.27	3.40	0.02	**< 0.001**
	Tree species	12	8.62	1.93	0.17	**< 0.001**
	N addition × tree species	12	4.52	1.01	0.09	0.426
	Residual	100	37.30		0.72	
N addition × tree clade	N addition	1	1.27	3.19	0.02	**< 0.001**
	Tree clade	1	1.33	3.34	0.03	**< 0.001**
	N addition × tree clade	1	0.56	1.40	0.01	**0.041**
	Residual	122	48.56		0.94	

Across all 13 tree species, *nifH* gene-based copy numbers did not differ significantly (*P* > 0.05) between N addition and control treatment, although slightly lower average values were observed under N addition (6.56 × 10^8^ copies $\:{\mathrm{g}}^{-1}$ dry weight) compared to the control (8.97 × 10⁸ copies $\:{\mathrm{g}}^{-1}$ dry weight). In *Populus*,* nifH* gene copy numbers were higher under N addition (2.53 × 10^9^ copies $\:{\mathrm{g}}^{-1}$ dry weight) compared to the control (9.02 × 10^8^ copies $\:{\mathrm{g}}^{-1}$ dry weight), although the difference was not significant (Figure S3).

### N Concentration and BNF in Deadwood

**Fig. 4:**
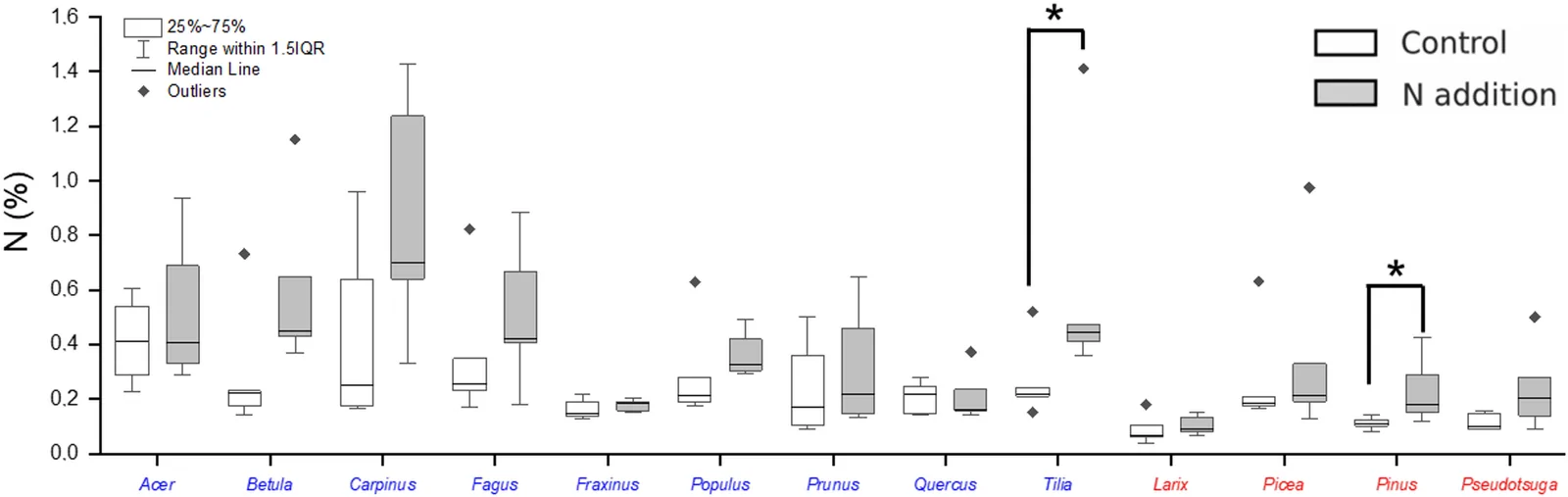
N concentration in deadwood of thirteen tree species subjected to two treatments (control, white; N addition, grey) after nine years of decomposition. Blue = broadleaved, red = coniferous. Significant effects of N addition within tree species are indicated by asterisks (*P* < 0.05)

**Fig. 5:**
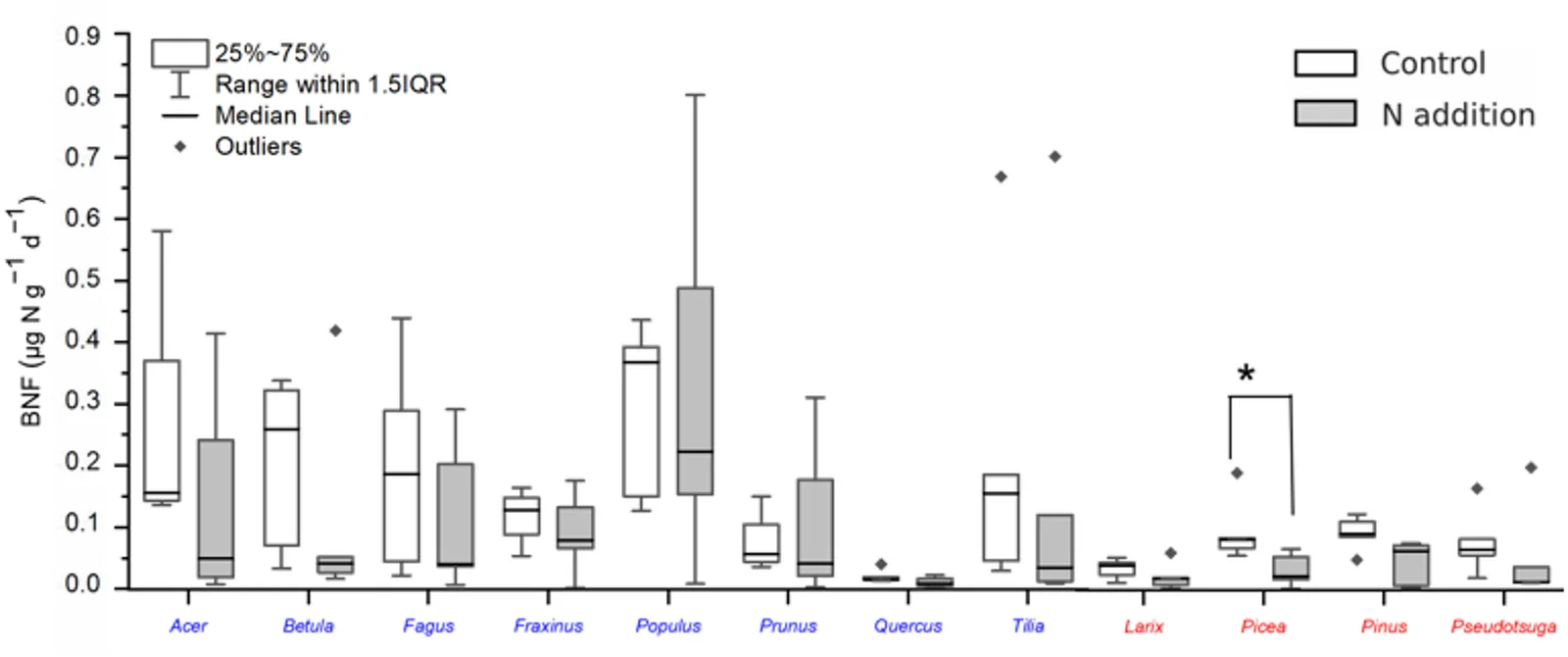
Biological nitrogen fixation (BNF) rates in deadwood from twelve tree species subjected to two treatments (control, white; N addition, grey) after nine years of decomposition. Blue = broadleaved, red = coniferous. Significant effects of N addition within tree species are indicated by asterisks (*P* < 0.05). BNF in deadwood of *Carpinus* was not determined

Mean N concentration in deadwood across all 13 tree species increased in both the control and N addition by 250% and 390%, respectively, between 2011 and 2020 (Table 2). At the end of the experiment, mean N concentration was 56% higher in the N addition as compared to the control. Deadwood N concentration was significantly affected by sampling year (*F* = 111.55, *P* < 0.001), N addition (*F* = 13.41, *P* < 0.001), and their interaction (*F* = 13.91, *P* < 0.001). N concentrations were similar between control and N addition treatments in 2011 and 2013. By 2020, however, N concentration had increased in both treatments and was significantly higher under N addition than in the control (Table 2). In 2020, N addition increased the mean N concentration in the deadwood of *Tilia* and *Pinus* by 130 and 109%, respectively, compared to the control (*P* < 0.05). Mean N concentrations were also higher in deadwood of other tree species in 2020, but the differences compared to the control were not significant (Fig. 4). The range of BNF rates in N-treated deadwood was found to be between 0.00 and 0.80 µg N g^− 1^ d^− 1^ compared to the control (0.01–0.67 µg N g^− 1^ d^− 1^). BNF rates in deadwood were lower under N addition compared to the control across all 13 tree species (− 43%), but the reduction was only significant for *Picea* (Fig. 5). A similar, though non-significant, trend of lower BNF rates under N addition was also present in broadleaved deadwood species, while the highest BNF rate was observed in *Populus* logs under N addition (0.80 µg N g^− 1^ d^− 1^). BNF was significantly correlated with variation in diazotrophic community composition in both control and N addition, whereas no significant correlation was observed with N concentration (Table S2). This relationship was significant for diazotrophic community compositions associated with broadleaved trees but not for those with coniferous trees (Table S2). 

**Table 2:** Mean (± SE) deadwood N concentration (%) under control and N addition treatments across three sampling points. Different lowercase letters indicate significant differences among sampling year x treatment combinations based on Tukey’s post-hoc test at *P* < 0.05. Means sharing the same letter are not significantly different

Sampling year	Treatment	*N* concentration
2011	Control	0.10 ± 0.01^a^
2011	N addition	0.10 ± 0.01^a^
2013	Control	0.11 ± 0.00^a^
2013	N addition	0.11 ± 0.00^a^
2020	Control	0.25 ± 0.02^b^
2020	N addition	0.39 ± 0.04^c^

### Relationships Between BNF, Mass Loss, nifH Gene Abundance, Diazotroph and Fungal Richness

BNF was not significantly correlated with mass loss, *nifH* gene abundance, and fungal richness in either tree clade or under either treatment (Table 3). However, BNF was significantly correlated with diazotroph richness in broadleaved logs (*R* = 0.47, *P* < 0.05) under the control treatment and across all logs in the control treatment (*R* = 0.37, *P* < 0.05) (Table 3). In coniferous logs subjected to N addition, BNF showed a weak, non-significant positive correlation with mass loss (*R* = 0.33, *P* > 0.05; Table 3). Furthermore, *nifH* gene abundance was significantly correlated with mass loss in broadleaved logs under both the control (*R* = 0.59, *P* < 0.05) and N addition (*R* = 0.46, *P* < 0.05) treatments, as well as across both treatments overall (Table 3). Additionally, a positive but not significant trend was observed between fungal richness and diazotroph richness under N addition in broadleaved logs.

**Table 3:** Spearman correlations between biological N fixation (BNF), *nifH* gene abundance, fungal richness, diazotrophic richness, and mass loss in deadwood from the control and N addition treatments in relation to tree species (broadleaved vs. coniferous). Correlations are significant at an adjusted *P* value < 0.05, and indicated in bold

	Control	*N* addition	Control Broadleaved	Control Coniferous	*N* addition Broadleaved	*N* addition Coniferous
BNF - nifH gene abundance	0.16	0.23	0.16	-0.20	0.31	-0.22
BNF - fungal richness	0.05	-0.03	-0.04	0.51	0.06	-0.01
BNF - diazotroph richness	**0.37**	0.24	**0.47**	0.03	0.36	0.04
BNF - mass loss	0.07	0.16	-0.07	-0.33	0.09	0.33
*NifH* gene abundance - fungal richness	0.03	0.21	0.24	-0.31	0.33	0.38
*NifH* gene abundance - diazotroph richness	0.25	0.15	0.19	0.36	0.27	-0.25
*NifH* gene abundance -mass loss	**0.48**	**0.40**	**0.59**	-0.09	**0.46**	0.10
Fungal richness - diazotroph richness	0.07	0.20	0.23	-0.18	0.30	0.12
Fungal richness - mass loss	-0.05	0.04	0.05	0.04	0.10	0.03
Diazotroph richness – mass loss	0.03	0.12	-0.03	-0.34	0.10	0.06

### Co-Occurrence Patterns of Fungal and Diazotroph Communities

**Fig. 6:**
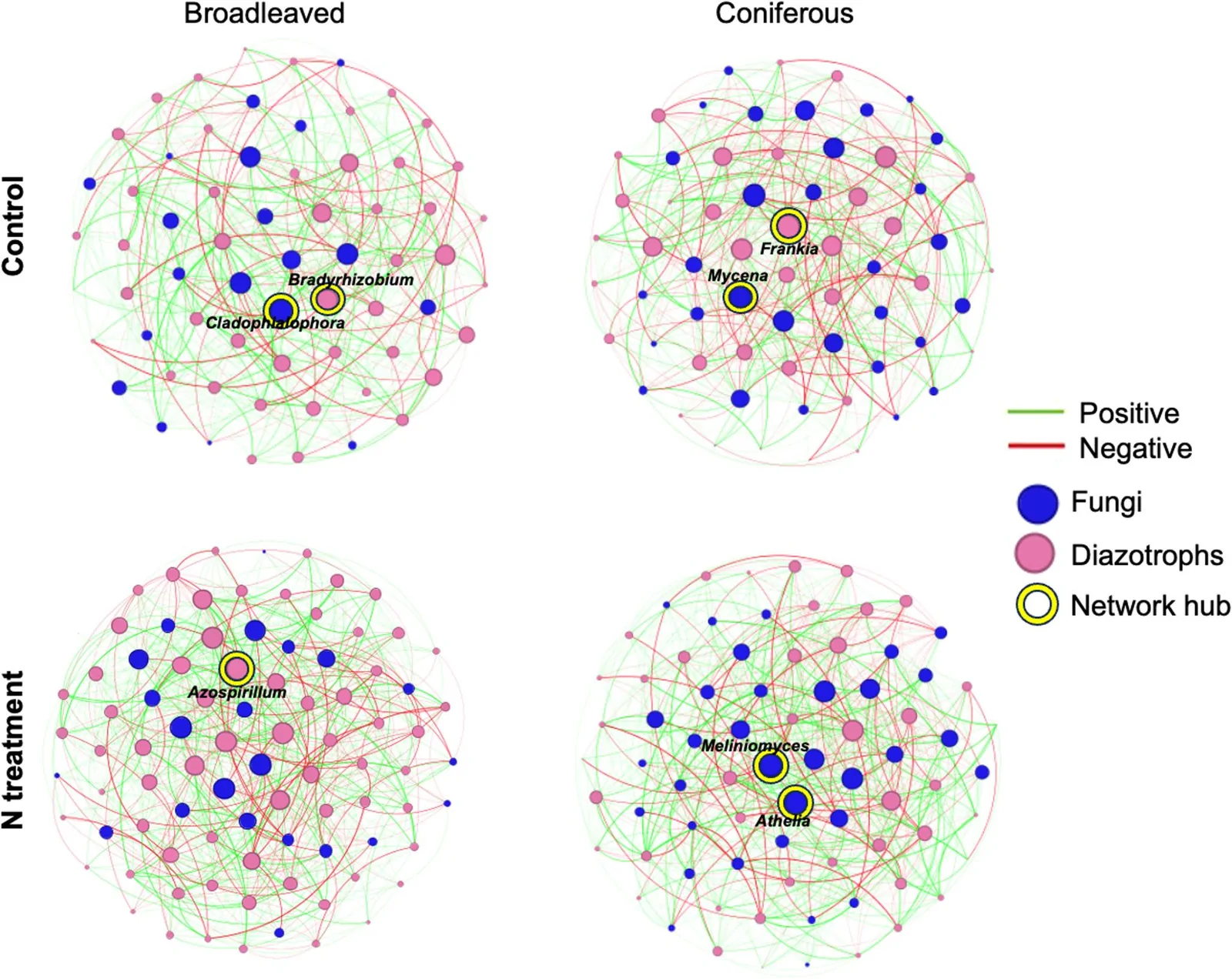
Co-occurrence networks of fungal-diazotrophic communities in deadwood from broadleaved and coniferous tree species subjected to two treatments (control, N addition) after nine years of decomposition. Nodes represent individual ASVs; edges represent significant correlations. The size of each node is classified based on the degree value

N addition resulted in a dense and interconnected network compared to the control treatment, with a higher number of nodes (taxa) and links (co-occurrence associations), a higher average degree (mean number of associations per taxon), and average path length as well as a lower average clustering coefficient for both broadleaved and coniferous logs (Table 4). The average clustering coefficient (the tendency of a taxon’s neighbours to also co-occur with each other) was lower under N addition, indicating that taxa were connected more broadly across the network rather than within tight local clusters. Together, these metrics suggest that N addition increased overall network connectivity and structural complexity while reducing local cohesion among co-occurring taxa. Under N addition, fungi and diazotrophs were more often positively associated, whereas negative associations were more common in the control treatment for both broadleaved and coniferous deadwood. We also found that the broadleaved and coniferous tree species had more modules (clusters of tightly interconnected taxa) under N addition than in the control treatment (Table 4). In addition, the taxonomic composition of microbial co-occurrence network was different between N addition and control treatment, for both broadleaved and coniferous logs (Fig. 6). For instance, in broadleaved tree species, *Cladophialophora* followed by *Bradyrhizobium* were placed at the central topological positions in the network of the control treatment, while diazotrophs (*Azospirillum*, ASV 20) showed the highest degree and betweenness centrality in the network of the N addition. In contrast, the reverse scenario was observed for coniferous tree species, where diazotrophs (*Frankia*, ASV 423) showed the highest degree and betweenness centrality in the control and fungi in the network of the N addition. Taxa with high betweenness centrality are considered keystone members whose removal would most disrupt community cohesion. 

**Table 4:** Topological characteristics of fungal-diazotroph networks in deadwood under control and N addition

	Nodes	Edges	Positive/Negative links (%)	Module number	Modularity	Average Degree	Average clustering coefficient	Average path length
Control								
Broadleaved	64	540	69/31	7	0.394	8.43	0.167	1.84
Coniferous	65	589	58/42	7	0.384	9.06	0.181	1.79
N addition								
Broadleaved	88	801	70/30	9	0.398	9.10	0.159	2.01
Coniferous	75	715	66/34	8	0.385	9.53	0.164	1.92
N addition Broadleaved Coniferous	88 75	801 715	70/30 66/34	9 8	0.398 0.385	9.10 9.53	0.159 0.164	2.01 1.92

## Discussion

Our study explored the impact of nine years of N addition on diazotrophic community composition and BNF in deadwood of 13 tree species. N addition led to significant shifts in diazotrophic community composition, with increases in *Bradyrhizobium* and losses of taxa like *Methylocapsa*, indicating that chronic N enrichment restructures the deadwood diazotrophic community rather than affecting all taxa uniformly. Further, N addition suppressed BNF in deadwood by 43% on average across the 13 tree species, although this reduction was significant only for *Picea*. This occurred despite no measurable changes in *nifH* gene abundance, indicating a decoupling between gene presence and function. Tree species had a strong influence on both community composition and richness, with coniferous logs showing more pronounced responses to N addition than broadleaved logs. Co-occurrence networks became more modular and interconnected under N addition, with central taxa shifting from fungi to diazotrophs in deadwood of broadleaved tree species and vice versa in coniferous tree species. Together, these findings suggest that long-term N deposition reshapes deadwood diazotrophic communities and their coupled ecosystem functions in a tree clade-dependent manner by increasing wood N concentrations, suppressing BNF, and accelerating gymnosperm-specific mass loss and fungal enzymatic activity in the same experiment [16]. These changes could potentially affect C turnover, nutrient cycling and the stability of forest ecosystem functions if N deposition remains high or continues to rise.

### Effects of N Addition on the Diazotrophic Community Composition

Our results indicate significant shifts in the diazotrophic community composition in response to N addition, with *Bradyrhizobium* becoming more abundant and *Methylocapsa* declining under N enrichment (Fig. 1). Members of both bacterial genera are slow-growing, methylotrophic Alphaproteobacteria that were frequently found in dead wood of the same [2] and other environments [18, 60]. Expression of methylotrophic enzymes involved in the utilization of C compounds generated during fungal wood decomposition has been reported [12], suggesting that both genera are also capable of consuming these C sources. In addition, this change of the diazotrophic community composition is consistent with the recent findings [8], which reported that high N loads produced greater shifts in diazotrophic community composition than low N loads in forest soils. In this study, we found that deadwood of different tree species harbored distinct diazotrophic communities, suggesting that tree species identity acts as an environmental filter shaping microbial assembly through substrate chemistry and microclimate. Within this context, N addition was associated with shifts in the relative abundances of specific diazotrophic genera, although these taxa remained low in overall abundance across tree species. This finding aligns with patterns reported in biocrust systems, where N addition similarly reshaped the relative representation of rare diazotrophic genera [61]. Taken together, elevated N levels altered diazotrophic community composition, with certain taxa, for example *Bradyrhizobium*, increasing in relative sequence read abundance, likely reflecting the decline of other taxa such as *Burkholderia*, *Corynebacterium*, *Serratia*, and *Sinorhizobium*, which were absent under N addition rather than an increase in the sequence read abundance of the remaining taxa. Diazotrophic communities maintain BNF under high N loads through compositional adjustments, with high N addition causing greater community restructuring than low N additions [8]. N addition significantly altered both diazotrophic richness and community composition in deadwood, with more pronounced effects observed in coniferous compared to broadleaved deadwood (Fig. 2 and S2). Additionally, tree species-specific responses were evident, as *Fagus*, *Prunus*, and *Picea* deadwood showed a significant increase in diazotrophic richness, while other species remained unaffected. N addition increased diazotrophic richness, particularly in coniferous deadwood (Fig. 2), is consistent with reports that N addition can alter diazotrophic diversity and community composition [62]. This increase in richness, however, was not accompanied by changes in *nifH* copy numbers or BNF rates, suggesting that the addition of N influenced the diversity of diazotrophic taxa present without stimulating their abundance or activity. Overall, these findings demonstrate that chronic N enrichment restructures deadwood diazotroph communities in a tree species and tree clade dependent manner.

### Impact of N Addition on N Concentration and BNF in Deadwood

Our study demonstrates that long-term N addition increased N concentration in deadwood, with a 56% rise observed by the end of the nine-year experiment (Table 2 ). This increase is likely due more to the addition and microbial incorporation of inorganic N rather than to BNF. Indeed, our results show that BNF rates were lower in N-treated logs, consistent with the well-documented suppression of diazotrophic activity under elevated N availability [63]. Differences in the increase in N concentration among tree species could be related to varying decay stages and the associated relative N enrichment [64]. For example, *Tilia*, with a 130% increase in N, is a tree species that decomposes relatively quickly, whereas *Pinus* (+ 109% N) decomposes relatively slowly. In *Pinus*, the higher lignin content slows fungal colonization and enzymatic breakdown [65], potentially limiting microbial N immobilization and resulting in a comparatively smaller N increase relative to *Tilia* over controls. Interestingly, the highest BNF rate following N addition was observed in *Populus* logs, with BNF reaching up to 0.80 µg N g^− 1^ d^− 1^. Nevertheless, the median BNF rate for this tree species was also lower than that of the control. The relatively high BNF activity in *Populus* could be attributed to high nutrient contents and pH that favor the abundance of diazotrophs [50]. Our results showed that BNF is still present in deadwood exposed to elevated N addition. The sum of background N deposition of about 15 kg ha y^− 1^ at the study region [49] and the N addition (40 kg ha^− 1^ y^− 1^) should be sufficient to completely cover the N requirements of deadwood-inhabiting organisms and to substitute the activity of diazotrophs. However, it is not clear to what extent the added ammonium-nitrate solution penetrated deadwood or was absorbed by deadwood organisms. Of the total added N over nine years, only a small fraction, < 0.001% was retained in the deadwood. 

### Relationships Between nifH Gene Copy Numbers, BNF, Mass Loss, Fungal and Diazotrophic Richness Under N Addition

N addition did not affect *nifH* gene copy numbers, but copy numbers were lower in coniferous than in broadleaved deadwood (Figure S3). This partially contrasts with our first hypothesis, which assumed reductions in *nifH* gene copy numbers under N addition, but supports our second hypothesis of tree species-specific responses. The fact that the N addition had no effect on *nifH* gene copy numbers, despite the decline in BNF rates, indicates that *nifH* gene abundance was not directly coupled to realized N fixation activity. Because *nifH* gene expression was not measured, it remains unclear whether the reduced BNF resulted from changes in *nifH* expression, post-transcriptional regulation, or direct inhibition of nitrogenase activity. This is consistent with findings from forest soils showing that diazotrophs can sustain or adjust their community composition under elevated N, even as fixation rates decline [8]. The lower *nifH* gene copy numbers in coniferous compared to broadleaved deadwood likely reflect the greater recalcitrance of coniferous wood characterized by higher lignin content and lower pH which constrains fungal colonization and the enzymatic breakdown of cellulose [29]. Since diazotrophs in deadwood depend largely on fungi for labile C substrates such as glucose released during cellulose degradation [29], however, diazotrophs may also use soluble wood-derived C or substrates released by other decomposer microorganisms. Thus, the absence of a significant relationship between diazotroph and fungal richness in coniferous logs under N addition may indicate that the diversity of the two groups was less closely coupled under these conditions. In contrast to the positive correlation observed in broadleaved logs (Table 3), suggesting a stronger association between fungal and diazotrophic community assembly. These contrasting patterns indicate that potential fungal-diazotroph associations depend on tree species identity and wood physicochemical conditions, although the underlying C transfer mechanisms were not directly assessed in this study. In broadleaved logs under N addition, we found a positive (though non-significant) trend between *nifH* gene abundance and BNF (Table 3), suggesting that where fungal–diazotroph interactions remain intact, diazotrophic abundance may still predict fixation potential. Conversely, the negative trend between BNF and *nifH* gene copy numbers in coniferous logs across both treatments suggests that, in these systems, a larger diazotrophic community does not translate into greater N_2_ fixation activity, likely because nutrient limitation and recalcitrant substrate chemistry constrain nitrogenase function regardless of community size. A weak or non-significant correlation between *nifH* abundance and BNF has been reported previously [50, 60], and our results suggest this decoupling is particularly pronounced in coniferous deadwood. In the broader literature, N addition has reduced *nifH* abundance under sustained high N loads [66], and BNF rates have similarly declined, although [8] showed that under chronic high N enrichment, community composition shifts can partially buffer BNF rates in forest soils. That we observed no such buffering in our experiment may reflect the more extreme nutrient limitations existing in deadwood relative to soil.

While greater diazotrophic richness might be expected to support higher BNF rates through increased functional diversity, richness alone does not determine N_2_ fixation activity. In broadleaved logs, a positive relationship between diazotrophic richness and BNF was maintained under both treatments (Table 3), suggesting that fungal-diazotroph interactions were maintained despite N addition. In contrast, no significant relationship was observed in coniferous logs, suggesting that N addition may suppress the activity of diazotrophic bacteria associated with coniferous trees as discussed in [67]. Although the positive trend between BNF and mass loss in coniferous logs under N addition was not statistically significant, it may reflect the release of other nutrients such as phosphorus and molybdenum during wood decomposition, which are co-limiting factors for nitrogenase activity and could support diazotrophic function even under elevated N availability. Increasing N availability through BNF may alleviate nutrient limitations, allowing decomposers to accelerate the breakdown of tough, lignin-rich wood. The positive relationship between *nifH* gene copy numbers and mass loss is more likely to reflect a response to decomposition than a driver of it, as deadwood breaks down, the accumulation of labile C substrates and fungal biomass may support larger diazotrophic populations, rather than diazotrophs actively accelerating decomposition.

### Influence of N Addition on Fungal and Diazotroph Interactions in Deadwood

Positive co-occurrences of diazotrophs and fungal communities suggest dense and facilitative species cooperation under N addition. Specifically, we observed a positive correlation between the fungal and diazotroph richness in broadleaved logs, which reflects the result of co-occurrence networks. N addition resulted in more positive connections between fungi and diazotrophs, contrasting with the negative connections observed in the control for both broadleaved and coniferous logs. This shift suggests that N addition likely strengthens fungal-diazotroph connectivity by selectively increasing the abundance of taxa within both groups that can coexist, thereby creating more opportunities for positive co-occurrence patterns [13, 68]. Associations between N-fixing bacteria and wood decaying fungi occur naturally in N-limited environments [13], suggesting these groups can form beneficial relationships. When external N reduces competition for this limiting nutrient, both groups may benefit from other complementary functions, leading to stronger positive associations. However, whether these patterns reflect mutualistic interactions or independent responses to elevated N cannot be determined from network analysis alone. Fungi dominate the breakdown of recalcitrant deadwood components through extracellular enzymes, while diazotrophs fix atmospheric N_2_ to meet their own metabolic demands. This fixed N becomes available to fungi through bacterial turnover and necromass mineralization, creating mutually beneficial relationships in N-limited deadwood [46]. This process enhances resource availability for both partners, as fungal enzymatic activity provides C sources that support diazotrophs [13]. Additionally, fungal breakdown of cellulose and hemicellulose releases labile C compounds that serve as energy substrates for diazotrophs, creating more favorable conditions for their colonization and activity in deadwood [12].

N addition alters the keystone taxa (network hubs) within fungal-diazotroph networks in a tree species-specific manner. In control logs, which had lower N than N added logs, diazotrophs occupied central network positions, for example, *Frankia* was a keystone taxon in coniferous control networks consistent with their functional role as the primary pathway of N input into N-poor deadwood. Under these conditions, diazotrophic activity supports fungal growth by making fixed N available through bacterial cell turnover, as discussed above. N addition fundamentally changes this dynamic, externally added N alleviates N limitation directly, reducing the dependence of the microbial community on BNF. N availability in the two scenarios is therefore not equivalent. In the controls, it is biologically mediated and tied to active diazotrophic communities, whereas in N-treated logs it is externally supplied despite lower diazotrophic activity. As a consequence, saprotrophic fungi become more active and assume greater network centrality, while diazotrophs lose their keystone status not because they are outcompeted, but because the ecological function they provided (e.g. N supply) is now fulfilled by N addition itself [69]. This pattern was specific to coniferous logs and was not observed in broadleaved logs. In broadleaved logs under N addition, *Azospirillum* emerged as a network hub (Fig. 6), maintaining dense connections with wood-inhabiting fungi. Members of the genus *Azospirillum* have frequently been found in association with saprotrophic fungi capable of decomposing broadleaved tree species and have been suggested to engage in mutualistic relationships with wood decaying fungi [13], which may contribute to their persistence and the maintenance of network connections under elevated N conditions [20]. In coniferous logs, *Frankia* gave way to fungi such as *Meliniomyces* and *Athelia* under N addition. consistent with saprotrophic fungi responding positively to relieved N limitation in a substrate where fungal activity is otherwise constrained by recalcitrant wood components [69]. Overall, these findings highlight a tree clade dependent reorganization of fungal-diazotroph associations in response to N enrichment. This distinction has implications for how deadwood microbial communities may respond to ongoing atmospheric N deposition, where increased N availability is externally driven rather than biologically mediated. The increased modularity observed under N addition, in both broadleaved and coniferous logs, suggests that N addition creates functionally distinct microbial clusters. This pattern is consistent with the idea that nutrient availability can drive specialization within microbial communities, leading to more compartmentalized interactions that support diverse ecological functions [13]. These results emphasize the importance of tree species identity in shaping how microbial communities and their interactions respond to nutrient enrichment in deadwood.

## Conclusions

Addition of ammonium and nitrate decreased BNF in deadwood, particularly in coniferous tree species, where N saturation makes the energetically costly process of N_2_ fixation less favorable. The decoupling of diazotrophic abundance from fixation activity suggests downregulation of nitrogenase function rather than loss of diazotrophic populations. N addition reorganized fungal-diazotroph network hubs in coniferous deadwood, shifting network centrality from diazotrophs in controls with lower N availability to saprotrophic fungi under N-enriched conditions, reflecting a shift from biologically mediated to externally supplied N. Even though atmospheric N deposition remains high in many regions of the world, only a limited inhibition of BNF and a change in the composition of microbial communities in coniferous deadwood are to be expected, which is likely to have only a minor impact on the C and N cycles in these forest ecosystems.

## Electronic Supplementary Material

Below is the link to the electronic supplementary material.

Supplementary Material 1 (572 KB)

## Data Availability

This work is based on data elaborated by Woodstock of the Biodiversity Exploratories program (DFG Priority Program 1374). The datasets are publicly available in the Biodiversity Exploratories Information System (http://doi.org/10.17616/R32P9Q). Data for N concentration (ID: 21809), Biological nitrogen fixation data (ID: 31332), mass loss data (ID: 31380), diazotrophic (ID: 32046) and fungal data (ID: 31524) are permanently available under the same link.
