# Supplementary material for: Chronic Nitrogen Deposition Alters Diazotrophic Community Composition, Reduces Biological Nitrogen Fixation, and Restructures Fungal-Diazotroph CO-Occurrence Networks in Deadwood
Source: Microb Ecol. 2026 Aug 1;89:153. doi: 10.1007/s00248-026-02849-5 (PMC13433720; doi:10.1007/s00248-026-02849-5)
Supplement: Supplementary file 1 [file 248_2026_2849_MOESM1_ESM.docx]

**Supporting information**

**Figures**

**Figure S1** Effect of N addition on the relative abundance of dominant diazotrophic taxa. Blue =N addition and red = Control. Different letters indicate the significant differences between control treatment and N addition.

**Figure S2** Non-metric multidimensional scaling (NMDS) ordination displaying diazotrophic community structure in deadwood of two treatments with (A) control and (B) N addition in relation to tree species (broadleaved = blue and of coniferous tree species = red).

**Figure S3** *nifH* gene abundance in deadwood from thirteen tree species subjected to two treatments (control, white; N addition, grey) after nine years of decomposition. Blue = broadleaved, red = coniferous.

**Figure S4** δ^15^N (‰) abundance in deadwood from thirteen tree species subjected to two treatments (control, white; N addition, grey) after nine years of decomposition. Blue = broadleaved, red = coniferous. Different letters indicate significant differences (*P* < 0.05) between control and N addition.

**Tables**

**Table S1** Effects of N addition, tree species or tree clade, and their interactions on diazotroph richness, *nifH* gene abundance, BNF, and deadwood N concentration. Significant variables *P* < 0.05 are shown in bold.

|  | Model | Effect | *F* | *P* |
| --- | --- | --- | --- | --- |
| Diazotroph richness | N addition × Tree species | N addition | 7.40 | **0.007** |
|  |  | Tree species | 4.50 | **<0.001** |
|  |  | N addition × tree species | 1.02 | 0.431 |
|  | N addition × Tree clade | N addition | 5.54 | **0.020** |
|  |  | Tree clade | 1.78 | 0.184 |
|  |  | N addition × tree clade | 0.84 | 0.360 |
| *nifH* gene abundance | N addition × Tree species | N addition | 0.32 | 0.567 |
|  |  | Tree species | 6.89 | **<0.001** |
|  |  | N addition × tree species | 1.06 | 0.401 |
|  | N addition × Tree clade | N addition | 0.21 | 0.644 |
|  |  | Tree clade | 5.32 | **0.022** |
|  |  | N addition × tree clade | 0.01 | 0.929 |
| BNF | N addition × Tree species | N addition | 13.62 | **<0.001** |
|  |  | Tree species | 3.95 | **<0.001** |
|  |  | N addition × tree species | 0.19 | 0.997 |
|  | N addition × Tree clade | N addition | 11.53 | **<0.001** |
|  |  | Tree clade | 8.48 | **0.004** |
|  |  | N addition × tree clade | 0.19 | 0.661 |
| N conc. | N addition × Tree species | N addition | 19.71 | **<0.001** |
|  |  | Tree species | 9.12 | **<0.001** |
|  |  | N addition × tree species | 0.80 | 0.645 |
|  | N addition × Tree clade | N addition | 14.02 | **<0.001** |
|  |  | Tree clade | 35.79 | **<0.001** |
|  |  | N addition × tree clade | 0.00 | 0.948 |

**Table S2** Goodness-of-fit statistics (R^2^), based on the ‘envfit’ function, for N concentration and BNF fitted to the two-dimensional non-metric multidimensional scaling (NMDS) ordination of diazotrophic community composition.

|  |  |  | Control | |  |  |  |  | N addition | |  |  |
| --- | --- | --- | --- | --- | --- | --- | --- | --- | --- | --- | --- | --- |
|  | **All tree species** | | **Broadleaved** | | **Coniferous** | | **All tree species** | | **Broadleaved** | | **Coniferous** | |
|  | r^2^ | *p* | r^2^ | *p* | r^2^ | *p* | r^2^ | *p* | r^2^ | *p* | r^2^ | *p* |
| N conc. | 0.02 | 0.56 | 0.00 | 0.99 | 0.12 | 0.33 | 0.05 | 0.28 | 0.01 | 0.81 | 0.13 | 0.30 |
| BNF | 0.38 | **<0.01** | 0.32 | **<0.01** | 0.27 | 0.05 | 0.18 | **<0.01** | 0.16 | **0.04** | 0.02 | 0.86 |
